# Identification of a novel *FGFR2-KIAA1217* fusion in esophageal gastrointestinal stromal tumours: A case report

**DOI:** 10.3389/fonc.2022.884814

**Published:** 2022-08-01

**Authors:** Yuehao Luo, Ying Wu, Xiaona Chang, Bo Huang, Danju Luo, Jiwei Zhang, Peng Zhang, Heshui Shi, Jun Fan, Xiu Nie

**Affiliations:** ^1^ Department of Pathology, Union Hospital, Tongji Medical College, Huazhong University of Science and Technology, Wuhan, China; ^2^ Department of Gastrointestinal Surgery, Union Hospital, Tongji Medical College, Huazhong University of Science and Technology, Wuhan, China; ^3^ Department of Radiology, Union Hospital, Tongji Medical College, Huazhong University of Science and Technology, Wuhan, China

**Keywords:** esophageal, quadruple *wild-type*, gastrointestinal stromal tumor, next-generation sequencing, *FGFR2-KIAA1217* fusion

## Abstract

**Background:**

Gastrointestinal stromal tumours (GISTs) rarely arise in the esophagus. The clinical course and treatment options for esophageal GISTs are poorly understood because of their rarity. In general, the mutation spectrum of esophageal GISTs resembles that of gastric GISTs. *Wild-type (WT)* GISTs lacking *KIT* and *PDGFRA* gene mutations occasionally occur in adults; primary esophageal GISTs are commonly *WT*.

**Case presentation:**

Herein, we report the case of a 41-year-old female patient who presented with a 1-week history of anterior upper chest pain. Chest computed tomography revealed a 3.7 cm × 2.8 cm × 6.7 cm soft tissue mass in the right posterior mediastinum adjacent to the esophagus. The patient underwent thoracoscopic mediastinal tumor resection and was subsequently diagnosed with an esophageal GIST. Neither *KIT* nor *PDGFRA* mutations were detected by Sanger sequencing; however, next-generation sequencing (NGS) identified an *FGFR2-KIAA1217* gene fusion in the tumor tissue. No relapse was observed in this patient during the 8-month treatment-free follow-up period.

**Conclusion:**

To the best of our knowledge, this report is the first to describe an *FGFR2-KIAA1217* fusion in a patient with a quadruple *WT* esophageal GIST. When *WT KIT/PDGFRA* GISTS are suspected, intensive genetic analysis is recommended, and obtaining a better molecular characterization of these tumours might reveal novel therapeutic avenues.

## Introduction

Gastrointestinal stromal tumours (GISTs) arise anywhere throughout the gastrointestinal tract and are seen most commonly in the stomach (40%–70%), small intestine (20%–40%), and colon and rectum (5%–15%) (1). Esophageal GISTs are extremely rare, accounting for 0.7% of all GISTs (2). Due to their rarity, clinicopathological data on esophageal GISTs are extremely limited, with only individual case reports or case series with small patient numbers available (3).

Most GISTs are characterized by oncogenic mutations in *c-KIT* (70% - 85%) or platelet-derived growth factor receptor-alpha (*PDGFRA*, 5%–15%) genes (4). The remaining 10-15% of GISTs lack *c-KIT* and *PDGFRA* mutations and are considered “*wild-type*” GISTs. These GISTs include *BRAF*-mutant GISTs, succinate dehydrogenase complex (SDH)*-*deficient GISTs, *KRAS*-mutant GISTs, and neurofibromatosis type 1 (NF1)-related GISTs.

The mutation status of esophageal GISTs has rarely been reported and is of increasing interest to researchers. Kang et al. found that the mutation spectrum of esophageal GISTs was similar to that of gastric GISTs in their case series, and most *KIT* mutations were detected in exon 11. Moreover, five (24%) of the 21 cases had *wild-type KIT* and *PDGFRA* genes, one of which was found to have the *BRAF* exon 15 V600E mutation by next-generation sequencing (NGS) (5).

Here, with the help of NGS detection technology, we found a rare *FGFR2-KIAA1217* fusion gene in the esophageal GIST of our patient, further expanding the *FGFR2* fusion variant spectrum.

## Case report

**Figure 1:**
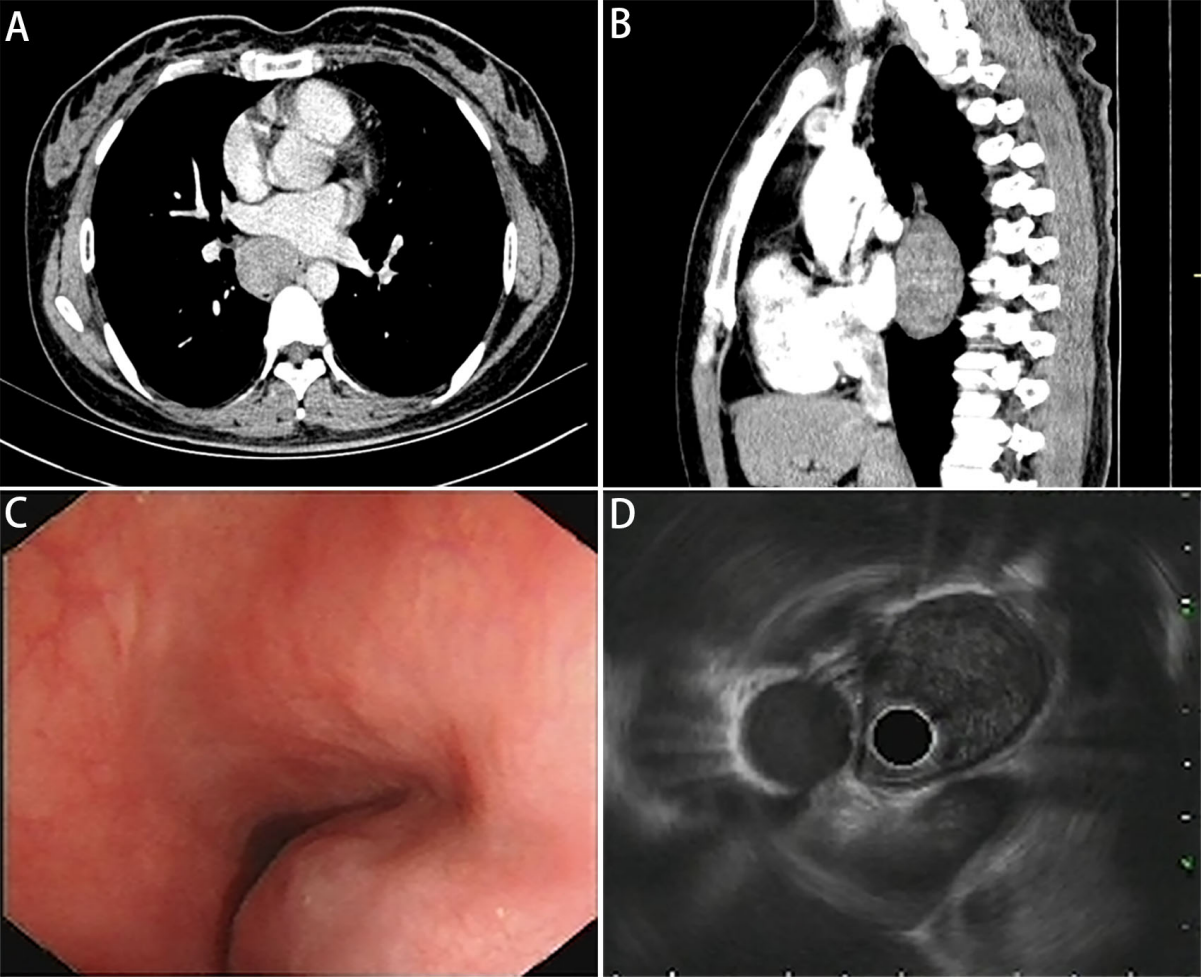
Images of pulmonary mediastinum CT and echogatstroscopy. **(A)** Transverse position and **(B)** sagittal position of pulmonary mediastinum CT show a mass located in the right posterior mediastinum approximately 37 × 28 × 67 mm in size, and the boundary with the adjacent esophagus is unclear. **(C)** The white light endoscopy view and **(D)** endoscopic ultrasound image show a 34.5 mm × 32.6 mm semispherical uplift with smooth surface mucosa and intraluminal growth pattern originating from the esophageal submucosal layer, with uniform hypoechoic change and a clear boundary.

**Figure 2:**
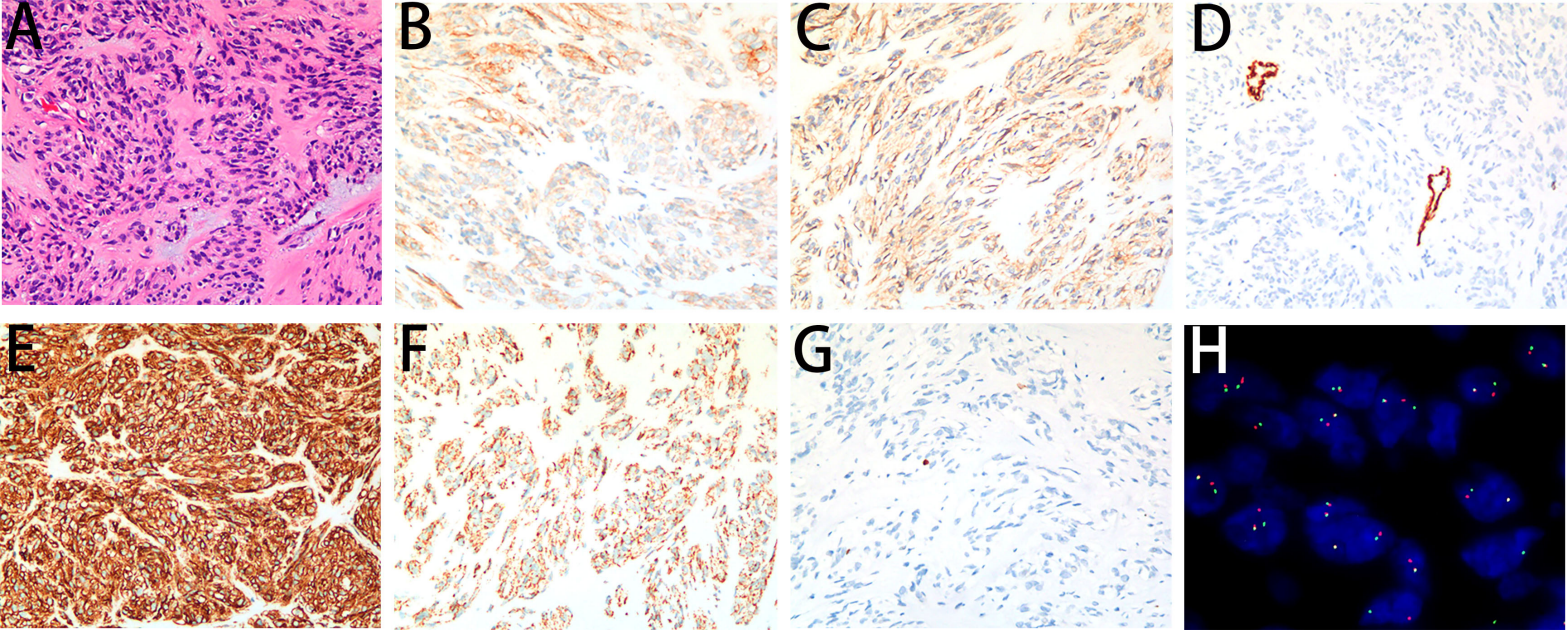
Microscopy images of the GIST. **(A)** The tumor demonstrated short spindle cells with red homogeneous stroma and pale blue mucus stained by H&E (200×). **(B)** The tumor cells showed an unequal positive cytoplasmic signal for CD117 (200×). **(C)** The tumor cells showed a weakly positive cytoplasmic signal for DOG1 (200×). **(D)** The tumor cells showed a negative cytoplasmic signal for SMA (200×). **(E)** The tumor cells showed a positive cytoplasmic signal for CD34 (200×). **(F)** The tumor cells showed a positive cytoplasmic signal for SDHB (200×). **(G)** The Ki-67 labelling index was 1% (200×). **(H)** A break-apart fluorescent *in situ* hybridization (FISH) assay found *FGFR2* translocation (100×).

A 41-year-old nonsmoker female was admitted to our hospital for chest pain that had persisted for over 1 week. Computed tomography (CT) imaging showed a soft tissue mass shadow in the right posterior mediastinum approximately 3.7 × 2.8 × 6.7 cm in size, and the boundary with the adjacent esophagus was unclear (**Figures 1A, B**). Endoscopic ultrasound examination showed a 3.5 cm × 3.3 cm submucosal eminence of the esophagus. The boundary remained clear with uniform hypoechoic changes (**Figures 1C, D**). Colour Doppler ultrasound of blood flow displayed little blood flow signals inside the mass. Ultrasonic elastography showed that the texture of the lesion was soft, and the strain ratio (SR) value was 4.91. The disease was initially diagnosed as a mediastinal mass. After the preoperative examination, the patient underwent mediastinal mass enucleation by video-assisted thoracic surgery (VATS). The tumor was completely resected macroscopically, and postoperative pathology supported the diagnosis of esophageal GIST. The mass (6.0× 4.0× 3.0 cm) showed a complete capsule and weave-like dimensional structure upon gross examination. The histological examination revealed that the tumor consisted of short spindle cells with red homogeneous stroma and mucoid degeneration (**Figure 2A**) with a mitotic index of < 5/5 square mm2. Thus, according to the modified National Institute of Health (NIH) criteria, the tumor was considered a high-risk GIST.

**Figure 3:**
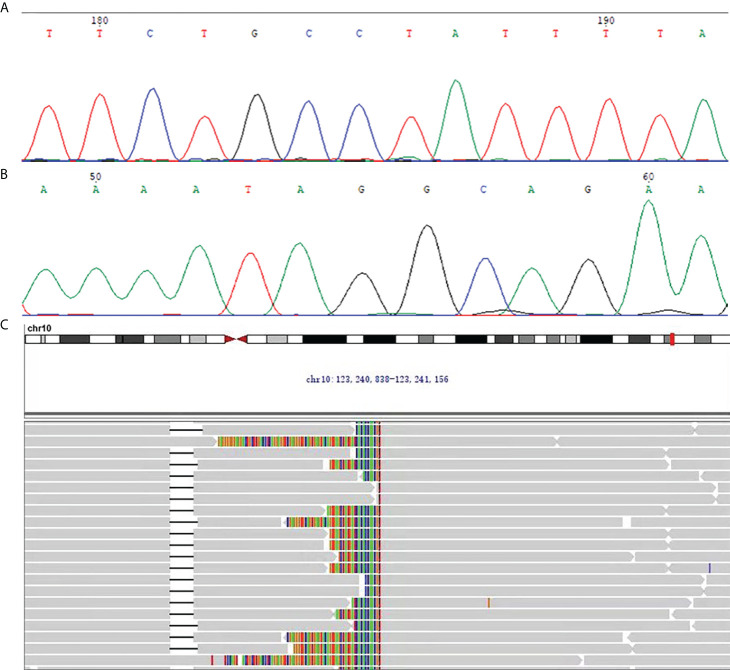
**(A, B)** The representative chromatogram of Sanger sequencing. **(A)** The forward sequencing result at exon 9 of *c-KIT* for the GIST in this case. **(B)** The reverse sequencing result at exon 9 of *c-KIT* for the GIST in this case. **(C)** NGS revealed the presence of the *FGFR2-KIAA1217* gene fusion (3.88% abundance in tissue).

On immunohistochemistry, the tumor cells were unequally positive for CD117 (**Figure 2B**), weakly positive for DOG1 (**Figure 2C**), negative for SMA (**Figure 2D**), strongly positive for CD34 (**Figure 2E**), and positive for SDHB (**Figure 2F**). The Ki-67 labelling index was 1% (**Figure 2G**). Sanger sequencing revealed no mutations in the exons corresponding to the *c-KIT* and *PDGFRA* genes, including exons 9, 11, 13 and 17 of *KIT* and exons 12, 14 and 18 of *PDGFRA* (**Figures 3A, B**). Next-generation sequencing (NGS) revealed the presence of an *FGFR2-KIAA1217* gene fusion (3.88% abundance in tissue, **Figure 3C**). A break-apart fluorescent *in situ* hybridization (FISH) assay confirmed the *FGFR2* translocation (**Figure 2H**). Follow-up was suggested for this patient. The patient did not experience relapse during the 8-month follow-up after surgery, and no further treatment was administered.

## Materials and methods

### Immunohistochemistry

The surgical specimens were fixed with 3.7% neutral formaldehyde and then routinely dehydrated, paraffin-embedded, sectioned at 4 μm, stained with HE and observed under a light microscope. Immunohistochemical staining was performed by the Envision two-step method. The primary antibodies were CD117 (Ventana Medical System, Inc., Tucson, AZ, USA; Clone: c-kit), DOG1 (MXB, Fuzhou, China; Clone: SP31), CD34 (Gene Tech, Shanghai, China; Clone: QBEnd 10), smooth muscle actin (SMA) (Gene Tech; Clone: 1A4), SDHB (ZSGB-BIO, Beijing, China; Clone: OTI1H6) and Ki-67 (MXB, Clone: MIB-5).

### Sanger sequencing

The Sanger sequencing method was used to detect c-KIT and PDGFRA gene mutations. According to the kit (c-KIT and PDGFRA gene mutation detection kit of Xiamen Aide Co., Ltd, Xiamen, China) instructions, genomic DNA was extracted from the samples. c-KIT exons 9, 11, 13, and 17 and PDGFRA exons 12 and 18 were amplified with polymerase chain reaction (PCR). After detecting the PCR products by gel electrophoresis, the PCR products were sequenced using ABI 3730XL DNA sequencers. The sequencing results were compared with the standard template sequences of the BLAST program within the CHROMAS software to identify the gene mutation loci.

### Next-generation sequencing

NGS was performed by Tongshu Gene (Shanghai, China). The OncoPanel consists of targeted sequencing of 556 cancer-related genes. The FGFR2 gene in the 556 panel for the detection of gene rearrangement and mutation were detected by DNA sequencing.

### Fluorescence *in situ* hybridization

Fluorescence *in situ* hybridization (FISH) was performed on 4‐μm‐thick FFPE sections using the Vysis dual-color FGFR2 break-apart probe kit (Abbott Molecular/Vysis, Des Plaines, IL) following the manufacturer’s instructions. The results were considered positive for FGFR2 when more than 30% of tumor nuclei had evidence of FGFR2 rearrangement in at least 100 tumor cells. The split signal was counted when the space between the two signals was larger than that of one signal.

## Discussion

Esophageal GISTs are a rare subtype, and limited data are available on the associated clinicopathologic features and clinical outcomes. The most common location of esophageal GISTs is the lower esophagus, followed by the middle esophagus; GISTs in the upper esophagus are rare (6). Esophageal GISTs are often found accidentally by oesophagoscopy or barium oesophagography (7). Dysphagia (36–51%) is the most frequent symptom in patients with esophageal GISTs, followed by weight loss (20%), chest pain (8–15%), and bleeding (1–10%) (1, 8). Here, the patient presented with atypical chest pain, and CT revealed a mass with a maximum diameter of 6.7 cm in the lower esophagus.

Spindle cell morphology is observed in 82.8% of esophageal GISTs, while 8–10% demonstrate epithelioid morphology and 8–10% mixed morphology (1). The vast majority of esophageal GISTs are positive for CD117, DOG-1 and CD34 (5). SDH-deficient cases are rare. Our patient’s tumor demonstrated short spindle-cell morphology and was highly positive for CD117, CD34 and DOG-1 expression. The current risk stratification systems are based on tumor size, mitotic counts per 50 high-power fields (5 mm2) and anatomical location (9). The risk for tumor recurrence was determined to be high according to the modified NIH criteria. Feng et al. reported that the majority of esophageal GISTs are classified into the high-risk category (70.83%) (1). On the other hand, when the risk classification system was established, only a few esophageal GISTs were included in the risk assessment, and the accuracy of these systems for determining the prognosis of patients with esophageal GISTs is unknown (10).

Data reported in the limited number of studies on esophageal GISTs suggest that the mutation spectrum of these GISTs resembles that of gastric GISTs. It was reported that the proportion of *KIT* exon 11 mutations is strikingly high in esophageal GISTs, usually involving codons 557 and/or 558 (5). However, our patient’s tumor did not harbor detectable *KIT* or *PDGFRA* mutations, as determined by Sanger sequencing. Next, we performed an in-depth targeted investigation into the genetic status of the so-called *KIT/PDGFRA wild-type* GIST by NGS to uncover putative alterations in frequently mutated genes that conventional molecular diagnostic approaches can miss. A novel *FGFR2- KIAA1217* fusion with a complete *FGFR2* protein kinase structure was identified in the absence of SDH/*RAS* pathway mutations. Therefore, our patient’s tumor was determined to be a quadruple *wild-type (qWT)* GIST. To date, only individual and rarely recurring alterations have been identified in this GIST subgroup, such as alterations in *ETV6-NTRK3*, *FGFR1*, *FGF4*, *MAX* and *MEN1 (* 11). Here, we report the first case of a *qWT* GIST harboring the novel *FGFR2-KIAA1217* variant, enriching the *FGFR2* fusion spectrum.

There is increasing interest in deregulation of fibroblast growth factor (FGF)/FGF-receptor (FGFR) signaling in different molecular subgroups of GISTs, which has emerged as a relevant pathway driving oncogenic activity. FGF/FGFR pathway alterations have been observed in *qWT* GISTs, including two activating missense mutations identified in *FGFR1* (p.K656E and p.N546K) and two gene fusions, *FGFR1–HOOK3* and *FGFR1–TACC1*. In *c-KIT*-mutated GISTs, the activation of the FGF/FGFR signaling pathway and crosstalk with the KIT receptor provide an alternative mechanism of imatinib resistance, representing a route for tumor cells to acquire secondary resistance to imatinib (12, 13). FGFR2 gain has been suggested as an additional potential mechanism associated with imatinib resistance (14).

Because of this rarity, the treatment of esophageal GISTs remains a matter of debate. Surgical resection is the only potentially curative treatment for localized GISTs. A small-sample exploratory study indicated that thoracoscopic enucleation of esophageal GISTs seems to represent a viable therapeutic option, as the postoperative morbidity/mortality is low and the oncological outcome is good for low-to-intermediate grade malignant tumours (15). Although imatinib treatment for GIST has been regarded as the paradigm of precision oncology, there is far from a consensus on the treatment of *qWT* GIST. Moreover, clinical studies of adjuvant imatinib therapy following resection of GISTs seldom involved esophageal GISTs. Therefore, more studies are needed to determine the effectiveness of adjuvant therapy for *qWT* esophageal GISTs.

Recently, pemigatinib and infigratinib, targeted oral therapeutic agents that are competitive inhibitors of FGFR1, FGFR2, and FGFR3, have been approved by the Food and Drug Administration (FDA) for locally advanced or metastatic cholangiocarcinoma with *FGFR2* fusion or rearrangements (16, 17). A recent clinicogenomic analysis of FGFR2-Rearranged Cholangiocarcinoma (FIGHT-202) suggest that patients with specific co-occurring alterations, especially in tumor-suppressor genes (including BAP1, CDKN2A/B, TP53, PBRM1, ARID1A, or PTEN) may have worse outcomes with Pemigatinib treatment. In the research, the authors found acquired FGFR2 mutations in all 8 patients analyzed at progression, consistent with previous reports of resistance to other FGFR inhibitors (18). Further, according to Varghese et al., polyclonal acquired mutations in the FGFR2 kinase domain were identified in acquired resistance to FGFR Inhibition by detectable circulating tumor DNA. Therefore, genomic profiling enables a deeper understanding of the molecular basis for response and nonresponse to targeted therapy (19).

So far, efficacy of the selective FGFR inhibitors in heavily pretreated phase 1/2 patients’ looks encouraging. A pivotal trial of derazantinib in advanced or inoperable FGFR2 gene fusion-positive intrahepatic cholangiocarcinoma reported an overall response rate of 20.7%, disease control rate of 82.8%. Estimated median progression-free survival was 5.7 months (20).In another phase I trial of advanced Solid Tumors harboring FGFR alterations, 58 patients receiving Debio 1347, there were 6 partial responses (3 with FGFR fusions) and 10 additional patients’ tumor size regressions of ≤30% (21). The development of targeted drugs for FGFR2 subtypes, such as bemarituzumab, an afucosylated monoclonal antibody against FGFR2b, and alofanib, a predominantly FGFR2-selective allosteric small-molecule inhibitor, is underway (22, 23).

Also, FGFR4, another member of FGFR family, has gradually been noticed. Indeed, while it is well known that its mutations are recurrent in several pediatric and adult rhabdomyosarcoma cases (24), recently its role has been proved also in gastric cancers, showing that mutationally activated FGFR4 may act as an oncoprotein, therefore supporting its therapeutic targeting (25). Therefore, FGFR-specific tyrosine kinase inhibitors may also provide a new approach to treating GISTs with FGF/FGFR pathway mutations.

It is worth noting that Caruso C et al. mentioned, Pharmacogenomics (PGx) biomarkers that can predict drug response play an important role in the improvement of molecular diagnostics in clinical routines in GIST patients. Association of SNP and outcome of GIST patients cured with imatinib and sunitinib was also highlighted by Kloth and colleagues. Thus, it is crucial to find novel prognostic biomarkers to stratify patients with improved risk for disease progression during imatinib therapy. Therefore, the potential role of FGFR2-KIAA1217 as pharmacogenomics biomarker in conventional** **chemotherapic protocol of GIST deserve further attention (26).

The clinical outcomes of esophageal GISTs from large case series studies indicated that 5-year disease-free survival (DFS) ranges from 57.0-65.3%. However, the overall survival (OS) results are controversial. Nakano et al. reported that the average time to recurrence was 40 months (5-year OS: 89%) and emphasized the need for long-term follow-up because recurrence occurred even 5 years after surgery (8). In contrast, Lott et al. reported that the 5-year OS of esophageal GISTs was 48.3%, suggesting a significantly worse prognosis than that of gastric GISTs. In our case, no recurrence occurred during the follow-up period of 8 months, and long-term follow-up was necessary.

This study is subject to several limitations. First, the duration of the follow-up was relatively short. Second, the therapeutic effects of the FGFR inhibitors or GIST targeted therapies were not clearly established.

## Conclusion

In summary, this report is the first to describe a *qWT* esophageal GIST patient with a novel, potentially targetable *FGFR2-KIAA1217* fusion. The case highlights the importance of a comprehensive genomic profiling approach able to detect all classes of genomic alterations, including uncommon gene fusions, to reveal potentially targetable somatic alterations for mutation-matched therapy selection.

## Data availability statement

The original contributions presented in the study are included in the article/supplementary material. Further inquiries can be directed to the corresponding authors.

## Ethics statement

Written informed consent was obtained from the individual(s) for the publication of any potentially identifiable images or data included in this article.

## Author contributions

XN and JF: conception and design of the work; acquisition and interpretation of data; revision of the manuscript critically for important intellectual content and scientific integrity. YL and YW: drafting of the manuscript and analysis of the patient’s data. PZ and HS: clinical management of the patient and provision of patient’s data. XC, BH, JZ and DL: elaboration and analysis of the pathology. All authors contributed to manuscript revision, read, and approved the submitted version.

## Funding

This work was supported by grants from the National Natural Science Foundation of China (No. 81773022 and 82072333); Natural Science Foundation of Hubei Province (No. 2020CFB808).

## Conflict of interest

The authors declare that the research was conducted in the absence of any commercial or financial relationships that could be construed as a potential conflict of interest.

## Publisher’s note

All claims expressed in this article are solely those of the authors and do not necessarily represent those of their affiliated organizations, or those of the publisher, the editors and the reviewers. Any product that may be evaluated in this article, or claim that may be made by its manufacturer, is not guaranteed or endorsed by the publisher.
